# A Neonate Presenting with Severe Dehydration: A Rare Case of Congenital Adrenal Hyperplasia with Salt Losing Crisis

**DOI:** 10.31729/jnma.8777

**Published:** 2024-10-31

**Authors:** Anita Lamichhane, Rekha Phuyel, Manish Upreti, Ramesh Khadka

**Affiliations:** 1 Department of Pediatrics, Lumbini Medical College, Palpa, Nepal

**Keywords:** *adrenal insufficiency*, *congenital adrenal hyperplasia*, *steroid 21-hydroxylase*

## Abstract

Congenital adrenal hyperplasia (CAH) is a rare autosomal recessive disorder caused by mutations in genes involved in cortisol biosynthesis in the adrenal gland. Depending on the enzymatic defect, the symptoms, signs, and laboratory findings differ. The most common form, accounting for more than 95% of cases, is caused by 21-hydroxylase deficiency. Delay in the diagnosis and treatment can lead to life-threatening adrenal crisis with hemodynamic collapse. We report a case of a five-day-old male neonate with congenital adrenal hyperplasia and salt-wasting crisis. The diagnosis was made after comprehensive assessment of clinical features and laboratory investigations. He was treated with hydrocortisone and fludrocortisone and was discharged after one week.

## INTRODUCTION

Congenital adrenal hyperplasia (CAH) is a rare autosomal recessive disorder caused by mutations in the steroidogenic enzyme genes involved in cortisol biosynthesis. The most common form of CAH, accounting for over 95% of cases, is caused by 21-hydroxylase deficiency, which results from a mutation in the CYP21A2 gene.^1^ Genital ambiguity in female newborns with CAH allows for early diagnosis, while males are usually asymptomatic at birth and are generally diagnosed after life threatening adrenal crisis or they die unsuspected.^2^

This report presents a unique case of a male neonate with CAH, emphasizing the crucial need for early diagnosis and intervention for timely management and prevention of complications.

## CASE REPORT

A five-days old male neonate presented to our emergency department with four days history of loose stools about 15 to 18 episodes a day, grade 5, non-foul smelling and not associated with mucous or blood; associated with decreased urinary output, fever, poor feeding, lethargy and weight loss. He was born at 36 weeks of gestation via normal vaginal delivery to a G2A1 mother with non-consanguineous marriage without any adverse perinatal events. His birth weight was 2200 grams (below tenth percentile for age). There were no antenatal risk factors or any genetic diseases in the family. Antenatal period was uneventful. The neonate cried immediately after birth. There were no risk factors for sepsis.

**Figure 1:**
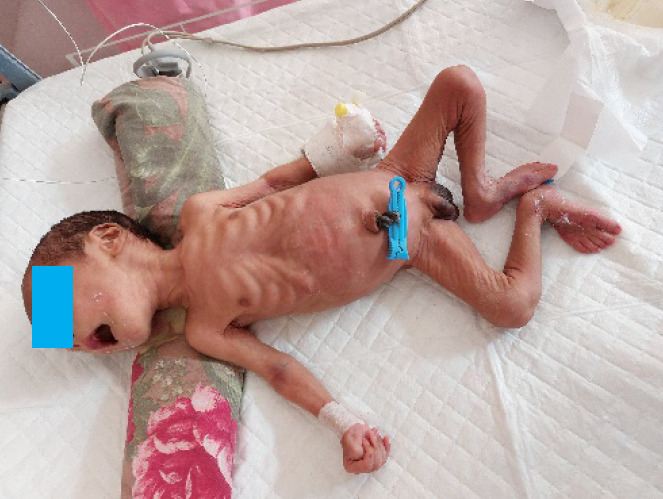
Showing the five days old neonate

**Figure 2:**
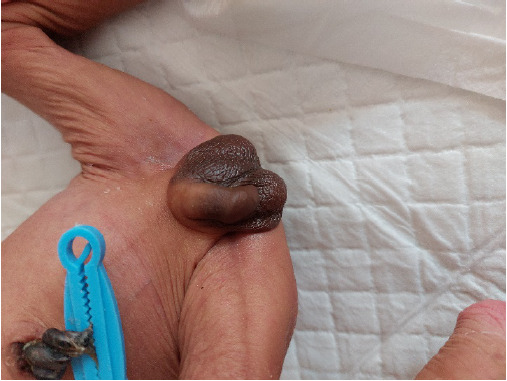
Showing the hyperpigmented scrotum of the neonate

On initial examination, he was dehydrated with sunken eyes with loose skin folds (Figure 1), lethargic with poor sucking. His weight was 1575 gms (below tenth percentile for age) with a loss of 28% form birth weight. His head circumference was 30 cm (below tenth percentile for age) and length was 48 cm (above fiftieth percentile for age). The baby was febrile with a temperature of 101 ^o^F, a pulse rate of 130 bpm, a blood pressure of 64/47 mm of Hg (> 50th centile) and oxygen saturation was 97% in room air. Respiratory, cardiovascular and central nervous system examinations were within normal limit. Genital examination revealed hyperpigmented skin over the scrotum (Figure 2).

On investigations, septic screening was negative with deranged electrolytes (Table 1). The stool test for reducing sugar was negative, and liver function tests were normal. X-ray and Ultrasonography of the abdomen did not reveal any abnormality. Initial blood gases showed metabolic acidosis. Hypoglycemia was managed with 10% dextrose, initially bolus was given @ 2ml/kg followed by continuous infusion of 10% dextrose with N/5 Normal saline to maintain the glucose level (GIR 6-8 mg/kg/min). He was managed accordingly as a case of hypernatremic dehydration with fluid therapy (20 ml/kg of 0.9% saline followed by maintenance fluid). Potassium was corrected with salbutamol nebulization and calcium gluconate. Despite initial correction, sodium and potassium concentration at 48-hours post-admission were 129 mEq/l and 7 mEq/l, respectively. So, a provisional diagnosis of Congenital Adrenal Hyperplasia (CAH) with salt-wasting crisis and pseudohypoaldosteronism (PHA) was made, and serum cortisol and 17 hydroxy progesterone levels were sent for testing.

**Table 1:** Laboratory investigation

Parameters	Result	Reference range
Haemoglobin	20.9 g/dL	14-24 g/dL
Haematocrit	64 %	44-70 %
Random blood sugar (RBS)	1.61 mmol/L	3-6.5 mmol/L
Serum Sodium	159 mEq/L	135-145 mEq/L
Serum Potassium	7.84 mEq/L	3.7-5.9 mEq/L
Blood Urea Nitrogen (BUN)	49 mg/dl	2.8-23.0 mg/dL
Serum creatinine	3.5 mg/dl	0.3-0.9 mg/dL

Serum cortisol [(23.11 mcg/dL), reference range=<5 mcg/dL] and 17 hydroxyprogesterone [(7768 ng/dL), reference range=<420 ng/dl) were both elevated. With significantly high level of 17 hydroxyprogesterone, a diagnosis of Congenital Adrenal Hyperplasia with salt-wasting crisis was made. The neonate was started on hydrocortisone intravenously (2mg/kg 6 hourly) and oral fludrocortisone. The blood electrolytes were monitored daily and gradually normalized at tenth day of admission. Due to unavailability of oral formulation of hydrocortisone, the baby was discharged on oral prednisolone (15 mg/kg/m^2^) and fludrocortisone acetate (0.3mg/day) and planned to follow-up after two weeks in the outpatient department. However, the baby was lost to follow-up.

## DISCUSSION

Congenital adrenal hyperplasia (CAH) is a group of autosomal recessive disorders caused by mutations in the steroidogenic enzyme genes involved in synthesis of cortisol from cholesterol in the adrenal gland. Several neonatal screening programmes conducted worldwide report the incidence of CAH to be approximately 1 in 10,000 to 1 in 20,000 live births.^3^ The most common cause of congenital adrenal hyperplasia, accounting for more than 95% of all cases, is due to a deficiency of 21-hydroxylase, an enzyme encoded by the CYP21A2 gene located on the short arm of chromosome 6.^1^ In CAH enzymatic defect in the corticosteroid and mineralocorticoid pathway shunts the steroid precursor to alternative derivatives like androgen and sex steroid. This leads to an increase in sex steroids alongside a decrease in cortisol and aldosterone synthesis.^4^

Disease manifestation depends on the severity of enzyme deficiency. Clinically, it is divided into classic forms, which include salt wasting and simple virilizing, and non-classic forms. Among the classic forms, approximately 75% of affected infants have the salt-wasting form, while 25% have the simple virilizing form. Females with classic 21-hydroxylase deficiency are exposed to excess androgens during the prenatal period leading to genital ambiguity characterized by an enlarged clitoris, labial fusion, and a common urogenital sinus. When the diagnosis is delayed, affected girls develop dehydration, hyponatremia, hypokalaemia and shock. Unless identified through neonatal screening, infant boys do not exhibit genital ambiguity, which could otherwise alert physicians. Instead, they may present with symptoms such as failure to thrive, poor feeding, lethargy, dehydration, hyponatremia, hypokalaemia, and shock, making the diagnosis is particularly critical in male infants.^2,5^

In developed countries, screening for 17-hydroxyprogesterone from heel-prick filter paper samples is routinely conducted for the screening of inborn errors of metabolism. This aids in early diagnosis and treatment of classic CAH.^7,8^

The treatment of CAH involves a multidisciplinary approach, incorporating paediatric endocrinologists, cosmetic surgeons, and psychiatrists. Oral hydrocortisone is recommended as the first-line replacement therapy in classic CAH. Additionally, fludrocortisone is administered alongside hydrocortisone.^6^ Females with ambiguous genitalia may require surgical intervention for the external genitals. For prenatal diagnosis in suspected pregnancies, amniocentesis or chorionic villous sampling can be performed to detect elevated 17-hydroxyprogesterone levels and mutations in the CYP21A2 gene. Once a prenatal diagnosis is confirmed, dexamethasone supplementation is administered to prevent in utero virilization of female fetuses with CAH.
